# The Effects of Exercise Training on Exercise Capacity and Vascular Function after Transcatheter Aortic Valve Implantation—A Pilot Study

**DOI:** 10.3390/jcdd10080343

**Published:** 2023-08-12

**Authors:** Luka Vitez, Matjaž Bunc, Borut Jug

**Affiliations:** 1 Department of Cardiology, Division of Internal Medicine, University Medical Centre Ljubljana, 1000 Ljubljana, Slovenia; 2 Faculty of Medicine, University of Ljubljana, 1000 Ljubljana, Slovenia; 3 Department of Vascular Diseases, Division of Internal Medicine, University Medical Centre Ljubljana, 1000 Ljubljana, Slovenia

**Keywords:** transcatheter aortic valve implantation, cardiac rehabilitation, exercise training, vascular function

## Abstract

Transcatheter aortic valve implantation (TAVI) improves event-free survival in patients with severe aortic stenosis, but patients’ exercise capacity remains poor after the procedure. Therefore, we sought to compare the effects of a supervised center-based exercise training program and unsupervised exercise routine on exercise capacity and vascular function in patients after TAVI. Patients were randomized to either center-based exercise training (12–24 sessions of combined aerobic and low-weight resistance training twice weekly for 8–12 weeks) or an unsupervised home-based exercise routine (initial appraisal with detailed recommendations and monthly follow-up). Exercise capacity (cardiopulmonary testing) and vascular function (ultrasonographic measurement of flow-mediated vasodilation (FMD) and arterial stiffness) were assessed at the baseline and after the study period. We included 23 patients (mean age of 81 years, 61% women), with higher-than-expected drop-out rates (41%) because of the coronavirus-19 pandemic outbreak. Exercise capacity improved over time, irrespective of the intervention group: 0.09 mL/min/kg increase in peak oxygen uptake (95% CI [0.01–0.16]; *p* = 0.02), 8.2 Watts increase in workload (95% CI [0.6–15.8]; *p* = 0.034), and 47 s increase in cumulative exercise time (95% CI [5.0–89.6]; *p* = 0.029). A between-group difference in change over time (treatment effect) was detected only for FMD (4.49%; 95% CI [2.35; 6.63], *p* < 0.001), but not for other outcome variables. Both supervised and unsupervised exercise training improve exercise capacity and vascular function in patients after TAVI, with supervised exercise training possibly yielding larger improvements in vascular function, as determined by FMD.

## 1. Introduction

Degenerative aortic stenosis is the most common valvular heart disease in developed countries [1]. It is associated with ageing and cardiovascular risk factors, confers high morbidity and mortality risk [2,3], and yields severe physical impairment due to a heavy symptom burden and exercise intolerance [4]. On the one hand, exercise intolerance can be explained by changes in cardiovascular hemodynamics: altered hemodynamics is a hallmark of aortic stenosis, with a reduced stroke volume and impaired pulse wave contributing to increased arterial stiffness, decreased wall shear stress, and impaired endothelial function [5]—all of which have an impact the individuals’ response to exercise [6]. On the other hand, physical limitation may be a direct result of severe aortic stenosis symptoms and frequent episodes of physical immobilization (e.g., during hospital admissions) [7], which may lead to a vicious circle of increasing physical inactivity and decreasing exercise tolerance.

Transcatheter aortic valve implantation (TAVI) provides an effective and safe treatment option for severe aortic stenosis, especially in vulnerable patients with multiple co-morbidities [8]. As compared to traditional surgical aortic valve replacement (sAVR), TAVI predominantly caters to older populations with multiple risk factors and co-morbidities [9,10]. Hence, TAVI corrects the central hemodynamic derangement of aortic stenosis (i.e., replaces the dysfunctional valve causing left ventricular obstruction) and improves survival, but it does not address other aspects of the aortic stenosis syndrome, such as patients’ frailty and deconditioning [11]. 

Exercise-based cardiac rehabilitation is a pivotal intervention for several cardiovascular diseases and improves exercise capacity, vascular function, and quality of life [12,13,14,15]. Retrospective observations of patients after TAVI who were selected for, and agreed to participate in, cardiac rehabilitation suggest that participation in rehabilitation programs is associated with improved event-free survival [16]. Prospective comparisons of TAVI and sAVR have also shown that cardiac rehabilitation improves walking distance (on the 6 min walk test, 6MWT) and daily living activities [17,18]. Disappointingly, however, randomized trials of exercise-based cardiac rehabilitation in patients after TAVI are scarce. One seminal randomized pilot study has shown that 8 weeks of exercise training improved participants’ peak oxygen uptake (VO_2peak_), muscular strength, and quality of life, when compared to usual care [19]. The effects of exercise training on other indicators of cardiovascular health, such as vascular function, remained elusive.

In the present study, we sought to compare the effects of a supervised center-based exercise training program and an unsupervised home-based exercise routine on exercise capacity, vascular function, and quality of life in patients after TAVI.

## 2. Materials and Methods

### 2.1. Study Population

We included consecutive patients after a successful TAVI implantation at the national TAVI referral center, the University Medical Centre (UMC) Ljubljana, Slovenia. Indications for TAVI were endorsed by the local Heart Team according to current ESC guidelines for valvular heart disease [20]. 

The inclusion criteria were as follows: TAVI implantation within 3 to 6 months, mobility (defined as more than 100 m on 6MWT), ability to attend 8–12 weeks of an outpatient cardiac rehabilitation program, and optimal medical treatment. 

The exclusion criteria were as follows: patients’ choice for TAVI albeit sAVR has been indicated by the local Heart Team; non-cardiac physical disability that would impede cardiac rehabilitation; a TAVI access site complication that required surgical treatment; unstable cardiovascular disease (e.g., New York Heart Association (NYHA) class IV, decompensated heart failure) or recent (<3 months prior to inclusion) cardiovascular event; active malignancy; severe chronic obstructive pulmonary disease or poorly managed asthma; severe peripheral artery, musculoskeletal, or central nervous disease that would impede bicycle exercise; and echocardiographic signs of bioprosthetic valve dysfunction (maximal velocity of more than 3 m/s, mean aortic gradient equal or more than 20 mmHg, aortic valve area under 1.2 cm^2^, or at least moderate paravalvular regurgitation [21]. 

### 2.2. Study Design

This prospective, single-center randomized controlled pilot study was conducted at the Center for Preventive Cardiology, Department of Vascular Diseases, UMC Ljubljana, Slovenia, from June 2019 to July 2020. Patient enrolment was terminated prematurely due to the coronavirus-19 (COVID-19) pandemic outbreak. Participants were randomly allocated into 2 groups (1 interventional and 1 control group) in a 1:1 ratio. Randomization was performed by the recruiting investigator (who was not the recruiting/treating physician to ensure concealed allocation) using adaptive (urn) randomization with sealed envelopes. All measurements were performed twice: at the baseline and after the study period (8–12 weeks).

### 2.3. Intervention

The supervised group underwent center-based cardiac rehabilitation, which consisted of an 8 to 12-week outpatient exercise training program with two visits per week (16–24 visits in total). The peak oxygen uptake (VO_2peak_) during a baseline cardiopulmonary exercise test (CPET) was used as the principal intensity parameter. The exercise sessions consisted of a 10 min warm-up, moderate endurance training (20 min of cycling at 40% VO_2peak_ with gradual progression towards a target intensity of 75% VO_2peak_ and 40 min duration), low-to-moderate-intensity resistance training (free weights and resistance bands), balance training, and a 10 min cool-down. Individual adjustments were permitted according to patient preferences and training progression under cardiovascular physical therapist supervision.

The unsupervised (control) group underwent a thorough baseline cardiovascular assessment, including CPET, and a 30 min informative session with a physical therapist. Regular exercise was recommended for at least 150 min per week (with timing and durations of 20–45 min at patients’ discretion) with the intensity based on CPET baseline results (target heart rate reserve of 40–75% and the adequate rate of perceived exertion). Follow-up was carried out monthly to appraise progression and/or recommend adjustment.

Our primary endpoint was defined as a change in VO_2peak_ assessed with CPET after intervention or after 8 weeks of a home-based exercise routine, respectively. 

### 2.4. Exercise Capacity and Muscle Strength

Aerobic exercise capacity was assessed using CPET. CPET was performed on a cycle ergometer Schiller CS-200. An adjusted ramp protocol was used with a 3 min warm-up without a workload, followed by gradual increase in workload by one tenth of the maximal estimated workload per minute, calculated on the basis of gender, age, and height. 

Patients were ECG monitored throughout the test and blood pressure measurements were taken every 2 min. During exercise, oxygen (O_2_) and carbon dioxide (CO_2_) flows were measured (VO_2_ and VCO_2_, respectively) using a mouthpiece connected to the device. We defined the ventilatory anaerobic threshold (AT) at the ratio between VO_2_ and VCO_2_ of 1.0. Participants were encouraged to give their maximal effort before they stopped cycling.

The following data were obtained from the test: the total time of the exercise test; peak workload in Watts (W); % of the expected peak workload (based on age, gender, and BMI); VO_2peak_; VO_2_ at AT; time of AT; respiratory exchange ratio (RER) calculated as the ratio of peak CO_2_ exhalation and VO_2peak_; O_2_ pulse; ventilatory equivalents for CO_2_ (VE/VCO_2_); basal heart rate (HR); maximal HR; heart rate reduction (HRR) at 1 and 3 min after exercise termination; and maximal systolic and diastolic blood pressure.

In addition, a 6MWT was performed using a standardized protocol with patients asked to walk between two marks on a 30 m distance under medical personnel supervision for 6 min.

Muscular strength was assessed with a hand grip test using a hydraulic dynamometer SH5001 (Seahan Corporation, Songak-myeon, Republic of Korea). Patients were asked to sit and use their stronger hand abducted and flexed in the elbow to 90 degrees. The grip of the dynamometer was adjusted according to the participants’ hand size. Three consecutive measurements were obtained and the highest was taken as the final result for further analysis. 

### 2.5. Vascular Function

All vascular function measurements were assessed using an Aloka Prosound a7 ultrasound machine. Flow-mediated dilatation (FMD), a marker of endothelial function, was measured on the right brachial artery and performed according to standardized practice by the same experienced investigator. Patients fasted and were advised to abstained from coffee, smoking, or exercise 3 h prior to measurements. After visualizing the brachial artery approximately 1 to 2 cm above the antecubital fossa, 3 arterial diameter measurements were obtained (d_baseline_). A forearm cuff was then inflated with pressure of 50 mmHg above the patient’s systolic blood pressure to maintain limb ischemia for 270 s. After 60 s of cuff deflation, 3 hyperaemic brachial artery diameter measurements were obtained (d_hyperaemia_). Finally, FMD was calculated as the percentage change in diameter using the following formula:

[(mean d_hyperaemia_ − mean d_baseline_)/mean d_baseline_] × 100.

Non-endothelial-dependent vasodilatation with nitro-glycerine was not assessed due to safety reasons in this frail population.

Carotid artery stiffness was measured on the right commune carotid artery through pulse wave analysis using an echo-tracking ultrasound. In the case of carotid artery murmurs or prior surgical or percutaneous procedures, measurements were not performed. The following parameters were included in the analysis: the beta (β) coefficient, pulse wave velocity (PWV), and augmentation index (AI).

Intra- and interobserver variability were assessed on 20 healthy subjects. The intraclass correlation coefficient for FMD measurements was 0.95. 

### 2.6. Self-Reported Health Status

Self-reported health status was assessed using a validated 36-item Short Form Survey (SF-36) and EuroQol 5-dimension, 5-level (EQ-5D-5L) questionnaire. SF-36 was developed at the RAND Corporation as a part of the Medical Outcomes Study. It consists of 11 questions referring to mental and physical functions, generating two combined parameters: a mental component summary (MCS) and physical component summary (PCS). Calculations were made using the German normative [22] with higher scores indicating an improved quality of life. EuroQol 5D (EQ-5D) is a preference-based measures questionnaire developed by the EuroQol Group [23]. It has been formally translated and validated into the Slovenian language [24,25]. It consists of a 5-dimension descriptive system, each containing 5 levels, and a final visual analogue scale (VAS). Calculated values range from 0 to 1, with higher scores indicating a better quality of life.

### 2.7. Laboratory Biomarkers

All patients’ venous blood samples were taken from the cubital vein after 30 min of rest in a supine position. Samples were withdrawn at the baseline and at the end of this study. Measurements included the glomerular filtration rate and B-type natriuretic peptides as safety markers to detect possible deteriorations in renal and/or cardiac function.

### 2.8. Statistical Analysis

Our primary endpoint was a change in functional capacity measured with the VO_2peak_ after the cardiac rehabilitation program. Power calculations suggest 36 patients would be required to detect a 1 mL/kg/min change in VO_2peak_ (a = 0.05, b = 0.2). Accounting for a drop-out rate of 10% in this elderly population, we decided to include 40 patients. Due to COVID-19 restrictions, inclusion was abruptly stopped after enrolling 39 patients. 

The normality of the data distribution was appraised visually and by the Shapiro–Wilk test. Descriptive statistics are expressed as means (with standard deviations) for normally distributed continuous variables, medians (with interquartile range) for non-normally distributed continuous variables, and as numbers (with percentages) for categorical variables. Between-group comparisons were carried out with a *t*-test for normally distributed continuous variables; Fisher’s exact test and Wilcoxon signed-rank test for non-normally distributed continuous variables; or χ^2^ tests for proportions. The statistical significance was set at *p* < 0.05. 

Between-group differences in change of outcomes (dependent variables) over the study period were estimated using linear mixed effects models (accounting for repeated measurements in each patient); patients were assigned as random effects, and time (pre vs. post), group (supervised vs. unsupervised exercise training), and time × group interaction were assigned as fixed effects. In the context of randomized trials, a significant time × group interaction suggests a significant effect of group allocation on outcome change over time (i.e., the treatment effect). However, given the randomization failure resulting in a significant between-group difference in the baseline body mass index (BMI), secondary/sensitivity analyses were carried out using multivariate adjustment with the BMI as a fixed covariate. Statistical analyses were performed with R version 4.2.1 (The R foundation for statistical computing, Vienna, Austria, 2022).

## 3. Results

**Figure 1:**
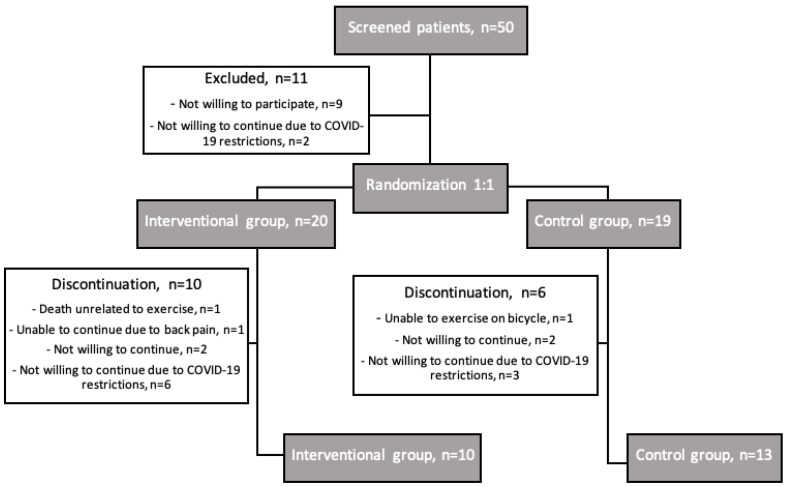
Patients’ study trajectory flowchart.

After screening 50 consecutive patients after TAVI, 39 were included in this study. Out of 20 patients randomized in the interventional group and 19 in the control group, 10 and 13 completed this study, respectively (Figure 1). We experienced a high drop-out rate of 41%, mostly related to the COVID-19 pandemic outbreak and restrictions. 

Patients were, on average, 81 ± 1 years old, 14 (61%) were women, and the mean time from TAVI to inclusion was 110 ± 4 days. Baseline characteristics were not statistically significant between groups, except for the BMI, which was significantly higher in the interventional group (*p* = 0.043). See Table 1.

The adherence to rehabilitation training was 100%, with five patients (50%) completing the program with 24 visits and five patients (50%) with 16 visits. All training sessions were safe, without reported side effects (e.g., dyspnoea, dizziness, palpitations, cardiac arrhythmias, chest pain). One death was reported in the interventional group during this study due to septic shock, which was deemed unrelated to exercise training (as reviewed by three treating physicians and one investigator). Laboratory safety endpoints—renal function and natriuretic peptides—did not differ significantly between groups: −8.1 mL/min/1.73 m^2^ (95% CI [−18.7; 2.6]; *p* = 0.136) for the estimated glomerular filtration rate (eGFR) and −0.14 ng/L (95% CI [−0.67; 0.39]; *p* = 0.606) for the log NT-proBNP levels, respectively.

**Figure 2:**
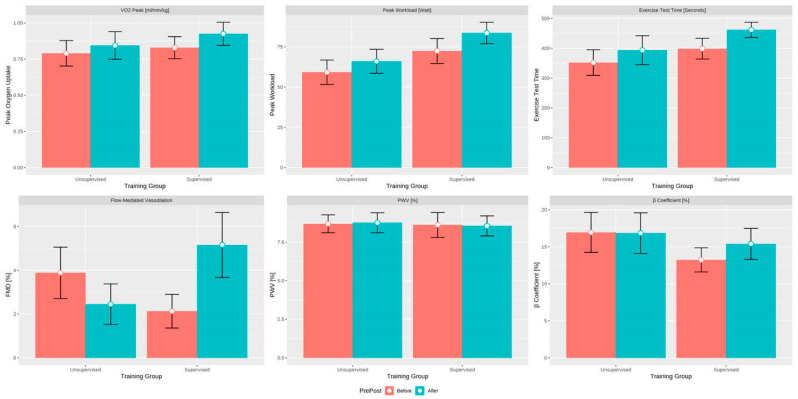
Exercise capacity and vascular function parameters before and after the intervention period. FMD = flow-mediated dilatation; PWV = pulse wave velocity.

Both groups improved their exercise capacity and vascular function over the study period. The cumulative exercise time significantly increased by 47 s (95% CI [5.0; 89.6]; *p* = 0.029), the exercise workload by 8.2 Watts (95% CI [0.6; 15.8]; *p* = 0.034), and the VO_2peak_ by 0.09 mL/min/kg (95% CI [0.01; 0.16]; *p* = 0.02) during the study period. A significant between-group difference in change over the study period was detected only for FMD (4.49%; 95% CI [2.35; 6.63]; *p* < 0.001)); statistical significance was retained after adjusting for the BMI (4.56%; 95% CI [2.44; 6.71]; *p* < 0.001). See Table 2 and Table 3 and Figure 2. 

Health status scores did not change significantly with respect to time or group. Study intervention was associated with a non-significant between-group difference in change of EQ-5D-5L TTO (time trade-off) health values—namely, an increase of 0.15 (95% CI [−0.01; 0.31]; *p* = 0.066) for the intervention group; the association was statistically strengthened after BMI adjustment (95% CI [0.0002; 0.31]; *p* = 0.05).

## 4. Discussion

Our study has shown that patients undergoing either supervised center-based exercise training or unsupervised home-based exercise routines after TAVI improve their exercise capacity and vascular function. We detected a between-group difference only for change in vascular function over the study period, as determined by improved FMD in the supervised training group, and no discernible change in health-related quality-of-life parameters over time or with respect to intervention. While marred by a higher-than-expected drop-out rate due to the COVID-19 pandemic, our findings may add insight into physiologic and clinical adaptations in post-TAVI patients—namely, an increase in exercise capacity over time, irrespective of center- or home-based exercise training and a possible added effect of supervised cardiac rehabilitation on vascular function.

Exercise training improves exercise capacity, which may be one of the strongest predictors of cardiovascular prognosis [26]. In our study, two approaches were employed: a supervised center-based exercise training program, and an unsupervised exercise routine with a detailed baseline appraisal and exercise recommendations with follow-up. Both approaches yielded significant improvements in exercise capacity parameters—namely, an 8.2 Watt increase in workload, a 0.09 mL/min/kg increase in VO_2peak_, and a 47 s increase in exercise testing time. However, our findings did not corroborate our initial hypothesis that supervised training would yield detectable differences in exercise capacity improvements. One randomized controlled trial previously reported that only patients in the exercise group significantly improved their exercise capacity and muscular strength, but the comparator—usual care—did not employ a structured recommendation [19]. Hence, our results may imply that exercise capacity in post-TAVI patients improves because a major limitation (i.e., severe aortic stenosis) is removed, thus allowing patients to improve physical activity and, in turn, exercise capacity. Additionally, the effect of a home-base exercise routine should not be underestimated and could have had a strong impact on our results. A recent observational pilot study showed it improves physical health and well-being, suggesting a fair solution to overcome the barrier of transportation to the medical facility [27]. As such, home-based rehabilitation in conjunction with newer types of digital health systems (e.g., telerehabilitation), could prove to be a valuable alternative to achieve better mobilization in patients after TAVI.

Conversely, we showed that supervised training improved vascular function (as determined by FMD) when compared to unsupervised exercise routines. FMD is a sensitive marker of endothelial function and a hallmark of vascular alterations in cardiovascular diseases [28]; as such, it has been extensively addressed in exercise trials in diverse populations [29,30,31,32,33] and has been shown to improve upon exercise training [6]. Several pathophysiological explanations have been proposed, such as exercise-induced improvements of endothelial function as a result of shear-stress-derived adaptations and increased nitric oxide (NO) bioavailability [33,34]. Our study findings therefore add to the growing body of evidence that FMD and endothelial function play a central role in the favorable cardiovascular effects of exercise training in diverse cardiovascular diseases. Parameters of arterial stiffness, however, did not change significantly. Arterial stiffness—as opposed to FMD—reflects morphological rather than functional changes and may be less sensitive to the effects of exercise, especially in populations with advanced impairments of vascular morphology, such as elderly individuals or patients with a high burden of risk factors and/or co-morbidities [35]. Interestingly, FMD decreased in the unsupervised group, a change most likely reflecting natural progression of endothelial disfunction with lack of sufficient exercise. 

Health status, as appraised with the SF-36 and EQ-5D-5L instruments, did not change significantly over time and/or with respect to intervention group. Our results, while suggesting a stability of health status over time, should be interpreted with caution. Previous studies have shown that participation in a cardiac rehabilitation program is associated with an improved health-related quality of life and increased health status measures [19,36,37]. In our study, however, the impact of the COVID-19 pandemic outbreak may have overshadowed any improvements brought about by the intervention, as several studies suggest that the pandemic has affected the quality of life in the general population [38,39,40,41,42]. Also, several health status measures have shown estimate directions that are in line with existing evidence but could not be statistically corroborated. Time trade-off scores for quality of life, for instance, decreased by −0.08 points (*p* = 0.167) over time but increased by +0.15 points (*p* = 0.061) if patients were randomized to supervised training. While the null hypothesis must be rejected in the present study, we urge further studies (not marred by unexpected shocks, such as the COVID-19 pandemic) to be undertaken and provide adequate answers with appropriate statistical certainty.

Our study has several limitations—first and foremost, the smaller-than-anticipated sample size due to the COVID-19 pandemic. This study was undertaken right before the outbreak of the COVID-19 pandemic, which reduced the accessibility of cardiac rehabilitation program and introduced safety challenges for patients at high risk for COVID-19 complications, such as those after TAVI. As a result, we experienced a lower-than-anticipated inclusion rate and a much higher than anticipated drop-out rate. This resulted in at least four methodological issues. Firstly, this study was underpowered to detect smaller magnitudes of difference in exercise capacity. Secondly, the likelihood of randomization failure increased (i.e., the BMI was higher in the intervention group and needed statistical adjustment). Thirdly, reasons for drop-out were very specific (COVID-19 pandemic) and likely affected the characteristics of patients who continued with this study as opposed to those who chose to withdraw. Fourthly, the heavily promoted local public health guidance for outdoor exercise during the pandemic has likely increased the exercise routines in the unsupervised group. These limitations notwithstanding, we believe our results merit reporting, as they add to the growing body of evidence on management options for the increasing post-TAVI patient population.

## 5. Conclusions

Both supervised and unsupervised exercise training improve exercise capacity and vascular function in patients after TAVI. In our study, only improvements in vascular function were significantly larger with supervised—as compared to unsupervised—exercise training. Our findings suggest that exercise training—be it supervised center-based or unsupervised home-based—improves indicators of cardiovascular health in patients after TAVI. Our study, however, should be regarded as a hypothesis-generating pilot study, as it was conducted during the COVID-19 pandemic and, as a consequence, included a smaller-than-intended and selected post-TAVI patient population.

## Figures and Tables

**Table 1:** Baseline characteristics of patients who completed this study. Data are presented as number (%), mean ± SD, and median (25th percentile–75th percentile).

Baseline Characteristics:	Interventional Group	Control Group	*p*
Participants number	10 (44)	13 (56)	
Age, years	81 (5)	82 (4)	0.617
Gender—female	7 (70)	7 (54)	0.363
BMI, kg/m^2^	30.9 (4.9)	26.3 (5.2)	**0.043**
NYHA Class			
I	1 (10)		0.178
II	8 (80)	13 (100)	
III	1 (10)		
6MWT, m	346 (77)	313 (68)	0.297
Hand grip test, kg	22 (3)	24 (6)	0.419
Systolic blood pressure, mmHg	158 (22)	163 (27)	0.568
Diastolic blood pressure, mmHg	82 (14)	75 (9)	0.138
Atrial fibrillation	3 (30)	3 (23)	0.537
Diabetes mellitus	3 (30)	4 (31)	0.663
Hypertension	9 (90)	12 (92)	0.692
Hyperlipidaemia	7 (70)	11 (85)	0.367
Coronary artery disease	4 (40)	9 (70)	0.164
History of acute myocardial infarction	1 (10)	3 (23)	0.404
Peripheral artery disease	1 (10)	1 (7)	0.692
History of cerebrovascular insult	1 (10)	1 (7)	0.692
Chronic obstructive pulmonary disease	3 (30)	1 (7)	0.200
Cardiac implantable device	4 (40)	3 (23)	0.337
**Echocardiography:**			
LVEF, %	57 (13)	55 (13)	0.676
Pulmonary artery systolic pressure, mmHg	35 (14)	40 (8)	0.247
**Laboratory:**			
Creatinine, mmol/L	95 (28)	80 (23)	0.178
eGFR, mL/min/10.73 m^2^	64 (23)	70 (14)	0.315
Haemoglobin level, g/L	135 (19)	134 (8)	0.901
HbA1c level, %	6.1 (1)	6.3 (0.9)	0.648
NT-proBNP, ng/L	573 (407–1025)	599 (222–2333)	0.973
Uric acid, mmol/L	366 (94)	334 (81)	0.401
Total cholesterol, mmol/L	5.2 (0.9)	4.4 (1.4)	0.137
HDL cholesterol, mmol/L	1.4 (0.4)	1.4 (0.4)	0.964
LDL cholesterol, mmol/L	3 (0.8)	2.3 (1.1)	0.107
Triglycerides, mmol/L	1.9 (1.2)	1.7 (0.8)	0.662
Myoglobin, mgl/L	61.9 (28.6)	61.9 (20.2)	0.872
CK, mkat/L	1.6 (0.7)	1.5 (0.8)	0.762
**Medications:**			
Aspirin	8 (80)	12 (92)	0.398
Oral anticoagulant	3 (30)	3 (23)	0.537
Angiotensin-converting enzyme inhibitor/angiotensin receptor blocker	8 (80)	11 (85)	0.596
Angiotensin receptor neprilysin inhibitor	1 (10)	0	0.435
Calcium channel blocker	2 (20)	4 (31)	0.463
Beta-blocker	7 (70)	13 (100)	0.068
Mineralocorticoid receptor antagonist	3 (30)	2 (15)	0.367
Furosemide	3 (30)	6 (46)	0.363
Statin	6 (60)	10 (77)	0.337
**TAVI:**			
Days after TAVI	101 (18)	118 (18)	0.081
Device used:			
Medtronic CoreValve Evolute R	4 (40)	5 (39)	0.157
Edwards Sapien 3	6 (60)	4 (31)	
Abbott Portico	0	4 (31)	
Valve-in-valve	2 (20)	1 (8)	0.398

**Table 2:** Baseline and final results for the observed groups (interventional/supervised center-based training vs. control/unsupervised home-based training). Data are presented as median (25th percentile–75th percentile). FMD = flow-mediated dilatation.

Exercise Testing:	Interventional Group			Control Group		
	Before, *n* = 10	Final, *n* = 10	*p*	Before, *n* = 13	Final, *n* = 13	*p*
Exercise time, s	418 (388–434)	466 (427–503)	**0.01**	356 (274–431)	369 (321–427)	0.092
Workload, W	75 (65–89)	82 (74–93)	**0.01**	59 (46–72)	66 (55–75)	0.11
VO_2_ peak, mL/min/kg	0.87 (0.66–0.98)	0.89 (0.80–1.08)	**0.049**	0.76 (0.64–0.91)	0.91 (0.72–0.98)	0.065
**Vascular Function:**						
FMD, %	2.10 (0.95–2.57)	4.55 (3.35–5.78)	**0.02**	3.00 (2.10–4.00)	2.30 (0.90–3.00)	0.069

**Table 3:** Between-group (interventional/supervised center-based training vs. control/unsupervised home-based training) differences for change in outcomes during the study period. Data are presented as coefficient (95% confidence interval). AT = ventilatory anaerobic threshold; VCO_2_ = carbon dioxide exhalation; VO_2_ = oxygen uptake; FMD = flow-mediated dilatation; AI = augmentation index; PWV = pulse wave velocity.

Exercise Testing:	Effect Size (Time)	*p*	Effect Size (Intervention)	*p*	Effect Size (Intervention × Time Interaction)	*p*
Exercise time, s	47.3 (5; 89.6)	**0.029**	46.7 (−28.3; 121.7)	0.222	15.9 (−47.1; 78.9)	0.620
Workload, W	8.2 (0.6; 15.8)	**0.034**	9.1 (−5.6; 23.9)	0.225	4.1 (−7.2; 15.3)	0.478
VO_2_ peak, mL/min/kg	0.09 (0.01; 0.16)	**0.020**	−0.01 (−0.18; 0.16)	0.925	0.01(−0.09; 0.12)	0.795
VO_2_ at AT, mL/min/kg	0.01 (−1.19; 1.2)	0.992	−1.07 (−3.39; 1.26)	0.369	0.18 (−1.56; 1.91)	0.842
VE/VCO_2_	−0.24 (−3.36; 2,88)	0.881	−0.6 (−4.38; 3.18)	0.757	0.3 (−4.62; 5.21)	0.906
O_2_ pulse, mL/beat	−2.32 (−5.95; 1.31)	0.211	−1.28 (−5.01; 2.44)	0.500	3.31 (−2.02; 8.63)	0.223
6MWT, m	1.8 (−22.8; 26.4)	0.886	32.4 (−21.4; 86.2)	0.238	24.7 (−11.9; 61.3)	0.186
**Muscular Strength:**						
Hand grip test, kg	−0.15 (−2.24; 1.94)	0.885	−1.31 (−5.17; 2.55)	0.507	2.48 (−0.68; 5.63)	0.124
**Vascular Function:**						
FMD, %	−1.45 (−2.87; −0.04)	**0.044**	−1.77 (−3.6; 0.07)	0.059	4.49 (2.35; 6.63)	**<0.001**
Β—coefficient	−0.22 (−2.5; 2.07)	0.854	−3.86 (−8.01; 0.29)	0.068	2.42 (−1.05; 5.88)	0.172
AI	−1.78 (−6.02; 2.47)	0.412	1.53 (−4.5; 7.55)	0.620	1.63 (−4.76; 8.03)	0.617
PWV, m/s	0.12 (−0.64; 0.86)	0.763	−0.17 (−1.37; 1.04)	0.788	−0.14 (−1.27; 0.99)	0.813
**Health Status:**						
EQ-5D-5L	−0.8 (−0.18; 0.03)	0.171	−0.09 (−0.18; 0.005)	0.064	0.15 (−0.01; 0.31)	0.066
SF-36 physical component	−61.8 (−275.9; 152.3)	0.571	−175.9 (−376.3; 24.4)	0.085	248.2 (−67; 563.5)	0.123
SF-36 mental component	3.4 (−7.3; 14.1)	0.532	2.4 (−13.3; 18.1)	0.765	−0.41 (−16.4; 15.5)	0.960

## Data Availability

Data will be available on request to the corresponding author.
